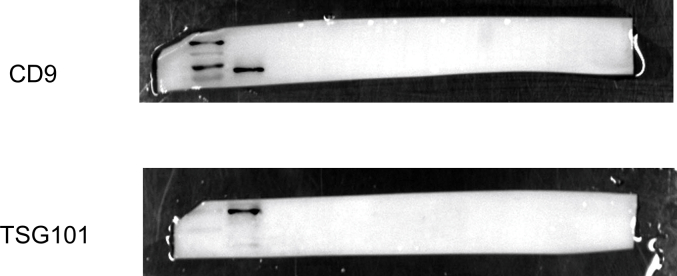# From bench to bedside: The promising value of exosomes in precision medicine for CNS tumors

**DOI:** 10.1016/j.heliyon.2024.e32376

**Published:** 2024-06-06

**Authors:** Mengjie Wang, Feng Jin, Xiaoguang Tong

**Affiliations:** a Clinical College of Neurology, Neurosurgery and Neurorehabilitation, Tianjin Medical University, Tianjin, 300070, China; b Qingdao Central Hospital, University of Health and Rehabilitation Sciences (Qingdao Central Hospital).266042, Qingdao, Shandong, China

**Keywords:** CNS tumors, Exosome, Transit platforms, Biotechnology, Precision treatment

## Abstract

Exosomes are naturally present extracellular vesicles (EVs) released into the surrounding body fluids upon the fusion of polycystic and plasma membranes. They facilitate intercellular communication by transporting DNA, mRNA, microRNA, long non-coding RNA, circular RNA, proteins, lipids, and nucleic acids. They contribute to the onset and progression of Central Nervous System (CNS) tumors. In addition, they can be used as biomarkers of tumor proliferation, migration, and blood vessel formation, thereby affecting the Tumor Microenvironment (TME). This paper reviews the recent advancements in the diagnosis and treatment of exosomes in various CNS tumors, the promise and challenges of exosomes as natural carriers of CNS tumors, and the therapeutic prospects of exosomes in CNS tumors. Furthermore, we hope this research can contribute to the development of more targeted and effective treatments for central nervous system tumors.

## Graphical abstract

The Promising Value of Exosomes in Precision Medicine for CNS Tumors(By Figdraw)

**Figure:**
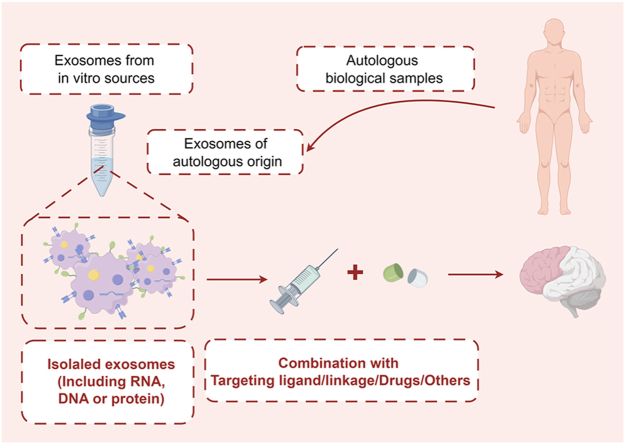

## 1 Introduction

Central Nervous System (CNS) tumors are a group of benign and malignant diseases originating from CNS tissue or structure. They are usually located in the intracranial or spinal canal and are among the most common CNS diseases. Notably, CNS tumors are clinically significant and have been associated with high disability and lethality risks.

Tumors of the CNS are divided into several groups: Brain tumors (Gliomas, glioneuronal tumors, and neuronal tumors), Choroid plexus tumors, Embryonal tumors, Pineal tumors, Cranial and paraspinal nerve tumors, Meningiomas, Mesenchymal and non-meningothelial tumors, Melanocytic tumors, Hematolymphoid tumors, Germ cell tumors, Sellar region tumors, and CNS Metastases,according to The 5th edition of the WHO Classification of Tumors of the Central Nervous System (WHO CNS5), 2021 [1].

Exosomes are naturally occurring small extracellular vesicles (EVs) that offer unique advantages as therapeutic delivery systems for precision treatment of brain diseases [2]. Exosomes have been found to play multiple roles within CNS diseases. These roles include key activities such as intercellular communication, waste disposal, inflammatory regulation, and neuroprotection [3]. Despite numerous studies, there is still a lack of clarity regarding the precise application of exosomes to the treatment of CNS diseases, particularly CNS tumors.

This review will detail the recent advances of exosomes in the diagnosis and treatment of various CNS tumors, the prospects and challenges of exosomes as natural carriers of CNS tumors, and the therapeutic perspectives of exosomes in CNS tumors, aiming to contribute to the study of precision treatment of CNS tumors and improvement of patient prognosis.

## 2 Exosome

### 2.1 Origin of exosomes

**Fig. 1:**
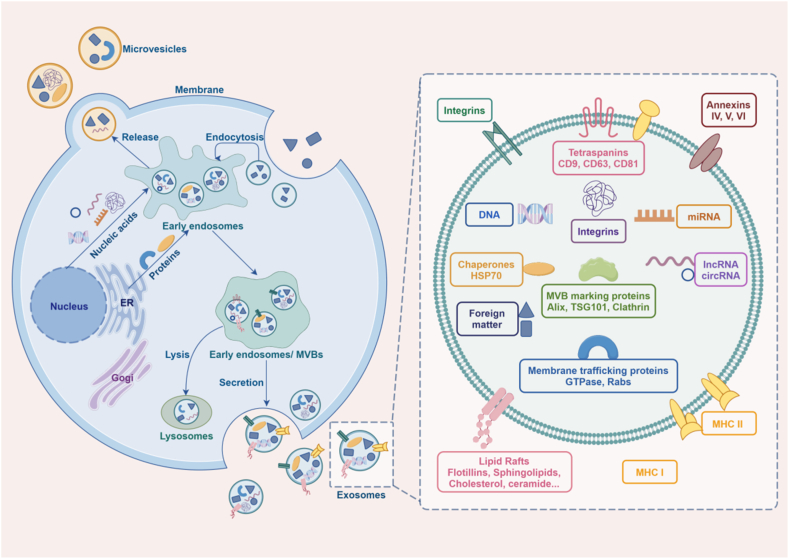
The secretion and contents of exosomes. Exosomes are released by fusion of multivesicular bodies and plasma membrane, and the lipid bilayer contains a large amount of genetic material, including DNA, miRNA, lncRNA, circRNA, etc. It carries a variety of proteins, including membrane transporter proteins, heat shock protein-specific marker proteins, and so on. These substances play an important role in tumor development. (By Figdrw).

Exosomes, tiny membrane-bound particles containing a variety of complex RNAs and proteins, are a type of EVs released into the surrounding body fluids upon the fusion of polycystic bodies and plasma membranes (Fig. 1). Exosomes are naturally present in body fluids, including blood, cerebrospinal fluid, saliva, milk, and urine, and are secreted by all culturable cell types [4]. The EV concept was first described in the 1940s. Rose Johnstone designated these EVs as exosomes [5]. Besides exosomes, there are two other types of EVs that could be differentiated from exosomes based on size and secretion patterns: Microvesicles (MVs) and apoptotic bodies [6]. Exosomes are released into the extracellular environment or different types of body fluids; this process was reported for different immune (dendritic cells as well as B-cells), epithelial, endothelial, and mesenchymal cells and was confirmed to be correlated with normal physiological processes. Different pathways play important role in the pathology of various diseases including tumor, cancer, Neurodegenerative Diseases [7,8].

Exosomes mediate cell-cell communication by activating intracellular signaling pathways. There are three primary modes of activation: Firstly, membrane proteins on exosome surfaces can activate signaling pathways in target cells by binding to their membrane proteins. Secondly, proteases in the Extracellular Matrix (ECM) can cleave exosomal membrane proteins into different fragments, which can act as ligands, binding to cell membrane receptors and consequently activating intracellular signaling pathways. Thirdly, the exosome membrane can directly fuse with the target cell membrane and non-selectively release the proteins, messenger RNA (mRNA, mRNA), microribonucleic acid (microRNA, miRNA), and other substances it carries [9].

### 2.2 Isolation and identification of exosomes

#### 2.2.1 Separate

**Fig. 2:**
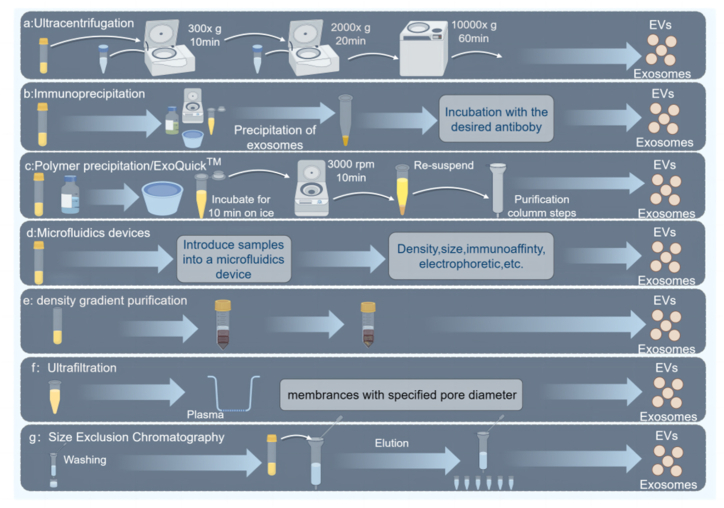
Separation method of the exosomes. A. Ultracentrifugation; B. Immunoaffinity; C. polymer precipitation; D. Microfluidics; E. density gradient purification; F. Ultrafiltration; G. size exclusion chromatography. (By Figdrw).

Typically, exosomes are extracted from isolated tissues and cell culture supernatants. The main exosome extraction methods include ultracentrifugation, density gradient purification, size exclusion chromatography, ultrafiltration, polymer precipitation, and immunoaffinity (Fig. 2).

#### 2.2.2 Identification

##### 2.2.2.1 Electric mirror

**Fig. 3:**
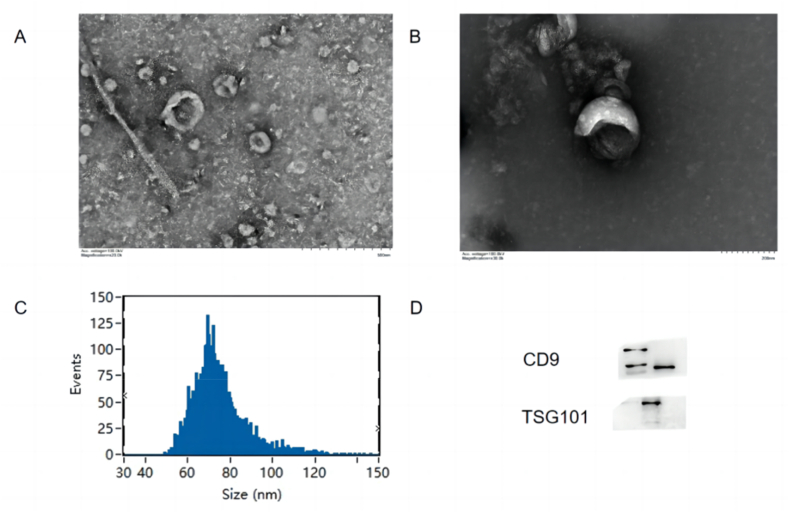
Identification of the exosomes. Exosome vesicles show cup or disk-like morphology under the electron microscope: A is the focal length of 500 nm, B is the focal length of 200 nm; C: the particle size distribution of exosomes is between 40 and 150 nm; D exosome-specific marker proteins (such as: CD9, TSG101) can be used for exosome identification.

Exosomes can be characterized and identified using a Hitachi brand HT-7700 transmission electron microscope. The operation steps are as follows: First, pipette 10 μL of the extract dropwise onto the copper mesh, allow it to precipitate for 1 min, and then remove the floating liquid with filter paper. Second, add 10 μL of uranyl acetate dropwise to the copper grid in the western region for 1 min, and then ventilate and allow to dry at Room Temperature (RT). Finally, set the electron microscope to 100 kV for detection and imaging. Most of the particles examined using electron microscopy were cup-shaped or spherical (Fig. 3 A, B).

##### 2.2.2.2 Particle size and concentration

The samples were examined using the NanoFCM brand N30E for particle size analysis and concentration determination. The steps were as follows: 1. Dilute 10 μL of the sample with PBS to 30 μL, and then use the standard to test the instrument performance before loading the sample; and 2. Complete the setup per the manufacturer's instructions. The particle size and concentration information will be available when the program finishes (Fig. 3C).

##### 2.2.2.3 Western blot

Although exosomes contain a variety of proteins, there are some common proteins in all exosomes, including those related to membrane transport and fusion (Rab, GTPases, and flotillin), those involved in the synthesis of multivesicular bodies (Alix and TSG 101), tetraspanins (CD9, CD63, CD81, and CD82), and cytoskeleton proteins (heat shock protein, actin, and tubulin). Therefore, exosomes can be identified by detecting these common proteins (Fig. 3 D).

### 2.3 Exosome contents

Exosomes primarily facilitate intercellular communication through the substances they carry. According to research, exosomes contain specific lipids, proteins, DNA, mRNA, and non-coding RNAs, which are critically involved in many physiological or pathological processes. The ExoCarta Exosome database (http://www.exocarta.org) currently has 9769 proteins, 3408 mRNAs, 2838 miNAs, and 1116 lipids identified in exosomes from different cell types and organisms (Fig. 1) [10].

## 3 Exosomes and CNS tumors

### 3.1 Exosomes and glioma

Exosomes and the molecules they carry could directly affect glioma progression and malignancy and are potential glioma biomarkers (Table 1). This approach facilitates early diagnosis through minimally invasive or non-invasive scans, enabling timely intervention and treatment. Typically, miRNAs are the most common of these markers. In a study by Lu et al., the level of miR-29a-3p in exosomes from blood plasma was significantly lower in glioma patients compared to healthy individuals, supporting its potential as a biomarker for the disease [11]. These biomarkers primarily influence glioma progression directly through gene expression or indirectly by activating specific signaling pathways. For example, GB cell exosome-derived miR-148a-3 pb specifically inhibits ERRFI1 expression and promotes tumor proliferation and angiogenesis via the EGFR/MAPK signaling pathway [12]. The biological markers used in glioma diagnosis and grading are generally exosome-bound and can be derived from glioma or circulating exosomes. He et al. found that glioma diagnosis and glioma-stage determination could be achieved based on miR-29a-3p expression in glioma cells and clinical data [13].

**Table 1:** A list of Representative exosome contents and related signaling pathways in glioma.

miRNA	Target gene/signaling pathway	Function/significance	References
miR-148a-3 pb	ERRFI1/EGFR/MAPK	Tumor proliferation angiogenesis	[14]
miR - 29a - 3p	PI3K/AKT/HIF-1α	Reduced glycolysis	[15]
miR-155-5p	(ACOT12) PCDH9/Wnt/β-catenin	Promote migration and invasion	[16][ 35]
miRNA- 26a	Phosphatase tensin homolog	Induces angiogenesis	[172]
miR-21, miR-222 and miR-124-3p	/	Biological marker	[173]
		Diagnostic grading	
miR-181d		Prognostic analysis	[174]
miR-301a	The AKT and FAK signaling pathways	Biological marker	[175]
		Promote both proliferation and invasion	
miR-221	DNM3	Biological marker	[176]
		Tumor resistance	
miR-25-3p	FBXW7	Prognostic marker cell multiplication	[177]
		Tumor resistance	
miR-1238	CAV1/EGFR	Tumor resistance	[178]
miR-375	CTGF-EGFR	Cell proliferation and invasion	[179]
miR3591-3p	JAK2/PI3K/AKT/mTOR	Biological markers	[180]
	JAK2/STAT3		
	MAPK		
circAHCY	miR-1294/Wnt/b-catenin/MYC/CTNNB1	Aberrant proliferation of normal human astrocytes	[17]
circCABIN1	Inhibition of miR-637	Promote reprogramming	[18]
		Tumor resistance	
circMMP1 (circ_0024108)	CircMMP1/miR-433/HMGB3 miR-29b-3p/DNMT3B/MTSS1	Promote both proliferation and migration	[19,20]
		Inhibition of apoptotic cell death	
circHIPK3	miR-421/ZIC5	Promote proliferation	[21]
		Tumor resistance	
circRNA 0001445	miRNA-127-5p/SNX5	Promoting glioma progression	[181]
circRNA BTG2 d	miR-25-3p/PTEN	Inhibition of the glioma progression	[182]
circ_0072083	miR-1252-5p/ALKBH5	Tumor resistance	[22]
Table 1. Continued			
circWDR62	miR-370-3p/MGMT	Tumor drug resistance and malignant progression	[23]
CircNFIX	miR-132	Promote tumor growth	[24]
		Tumor resistance	
		Prognostic marker	
circKIF18A	FOXC2	Participate in angiogenesis	[183]
RNU6-1	/	Biological markers	[184]
		Independent predictors	
L1CAM	Integrins and the fibroblast growth factor receptors(FGFRs)	Movement, proliferation, and invasion	[25]
lncRNA-ATB	Inhibition of the microRNA (miRNA or miR) -204-3p in a Argonaute 2 (Ago 2) -dependent manner	To promote both migration and invasion	[185]
lncRNA ROR1-AS1	miR-4686	Promote the proliferation, migration, and invasion	[186]
linc-CCAT2	/	Promote angiogenesis	[187]
		Inhibition of endothelial cell apoptosis	
SNHG16	TLR7/MyD88/NFκB/c-Myc	Promoting glioma progression	[188]
SBF2-AS1	The ceRNA of miR-151a-3p	Remodeling the microenvironment	[189]
		Tumor resistance	
MIF	TIMP3/PI3K/AKT	Tumor resistance	[127]
FASN	/	Biological markers of malignancy	[190]

Besides affecting glioma cell proliferation and apoptosis, the exosome-derived miRNA promotes glioma development by influencing its invasion and migration. Research has revealed that exosomal miR-155-5p significantly downregulated Acetyl-CoA Thioesterase 12 (ACOT12) and reduced the therapeutic effect of ACOT12 in inhibiting glioma [26]. Furthermore, exosomal miR-155-5p can directly target PCDH9 expression and enhance glioma migration and invasion by activating the Wnt/β -catenin signaling pathway [27]. It has been reported that EMT can promote glioma development by enhancing cell invasion and migration [28]. Additionally, it was found that Glioma Stem-like Cell (GSC)-released exosomes contain significantly higher miR-155-5p levels and can be horizontally transferred to the surrounding glioma cells, promoting EMT progression and accelerating glioma progression and invasive growth [26].

Circular RNA (circRNA) is a special circular non-coding RNA molecule and one of the critical exosome-bound molecules. It basically functions by influencing miRNA expression. High circRNA expression in brain tissue can promote glioma proliferation, immunosuppression, angiogenesis, invasion, migration, and therapeutic resistance by sequestering specific or competitive miRNA inhibition [29,30]. Exosome-derived circAHCY sequesters miR-1294 expression, activates the Wnt/β-catenin signaling pathway, upregulates MYC, enhances CTNNB1 transcription, and triggers the abnormal proliferation of normal human astrocytes [31]. Meanwhile, multiple studies have also demonstrated that CTNNB1 expression is closely related to glioma occurrence and progression [32]. For example, exosomal circCABIN1 competitively inhibited miR-637 expression, blocked the inhibitory effect of miR-637 on OLFML3, and promoted the reprogramming as well as glioma cell resistance to Telozolomide (TMZ) [28]. Furthermore, serum exosomal circMMP1 (circ _ 0024108) enhanced glioma cell proliferation and migration and inhibited apoptotic cell death through the CircMMP1/miR-433/HMGB 3 axis/CircRNA_104948 and miR-29b-3p/DNMT3B/MTSS 1 axis [33,34].

Moreover, circRNA can exacerbate glioma resistance to therapeutic drugs through diverse mechanisms, complicating treatment efforts and hastening disease advancement. For example, exosomal circHIPK3 promotes tumor cell proliferation through the miR-421/ZIC 5 pathway, while increasing resistance to TMZ [35]. Exosomal circ _ 0072083 can regulate ALKBH5 through miR-1252-5p and increase TMZ resistance in glioma [36]. Furthermore, glioma cells that have shown TMZ resistance will show a high expression of exosomes along with the molecules they carry, further affecting glioma's drug sensitivity. Exosomal circNFIX and circ _ 0072083 levels were significantly elevated in patients with TMZ-resistant glioma [34,37]. It was found that circWDR62 was upregulated in both the TMZ-resistant glioma cells and TMZ-resistant glioma cell-derived exosomes, and that it promotes TMZ resistance and malignant glioma progression by targeting the miR-370-3p/MGMT axis [38]. Si et al. discovered that Heparanase regulates circ _ 0042003 expression in exosomes of TMZ-resistant U251 cell lines, thereby affecting glioma cell resistance to TMZ and sensitizing U251 cells to the drug after knockdown [39].

Other molecules carried in exosomes may also influence the occurrence and progression of glioma. Duthika and colleagues performed a proteomic analysis of glioma-derived exosomes and found that five genes [Annexin A1 (ANXA 1), Actin-Associated Protein 3 (ACTR 3), Integrin- β 1 (ITGB 1), Insulin-like Growth Factor 2 Receptor (IGF2R), and Programmed Cell Death 6 Interacting Protein (PDCD6IP)] were highly expressed in GBM tumors than normal brain tissue-derived exosomes. These genes' expressed proteins have been implicated in actin polymerization and endosomal sorting and have shown a significant correlation with GBM cell invasion. As a result, they can be used as potential EV biomarkers for predicting the survival of GBM patients [40]. Connexin 43 (Cx 43), the most common linker protein in glioma cells, is highly expressed in glioma-derived exosomes and participates in hypo-exos-mediated glioma angiogenesis by promoting HUVEC proliferation, tube formation, and migration [[14], [41], [42]]. High Cx 43 expression levels were associated with resistance to TMZ by glioma cells. Additionaally, Cx 43 was shown to be significantly expressed in TMZ-resistant U251r cells and rExo. The rExo enhanced U251s cells’ TMZ resistance, colony formation, and migratory ability [43]. The immunoglobulin superfamily protein L1CAM (L1, CD171) is well-known to promote neuronal migration, differentiation, and axonal guidance during development [15]. Furthermore, the pseudogene Transmembrane Protein 198B (TMEM198B) is highly expressed in glioma tissues and cell lines and influences glioma progression by regulating lipid metabolic reprogramming [16]. The presence of trkb-containing exosomes restores cell proliferation, promotes Endothelial Cell (EC) activation, and promotes glioma development [44]. Moreover, exosomes carrying protein ligands can play an important role. Wang et al. found a unique protein-ligand in glioma patients that combined with dendritic cells in the cerebral spinal fluid (TIM 3 receptor in dc), inhibiting dc antigen recognition, processing, and presentation, leading to the failure of t-cell-mediated anti-tumor immune response, and exhibiting lasting anti-tumor characteristics [45].

Exosomes originating from various sources can play a role in glioma progression. Pace et al. found that the secretion of small l1-modified exosomes by GMB cells notably accelerated the migratory speed of three human glioma cell lines (T98G/shL 1, U-118 MG, and primary GBM cells), resulting in substantial GMB cell proliferation and GBM cell invasion [46]. The circulating exosomes’ DNA contains modulated exomes, exons, non-coding genomic DNA (gDNA) and chromosome sequences, as well as various regulatory genes and transposable element DNA sequences related to brain tumor secretory exosomes, including SOX 2, EGFR, MGMT, PTEN, TP 53, CDK 4, ERBB2, MDM 2, and so on. The regulatory methods include insertion, deletion, amplification, mutation, genomal rearrangement, chromosomal deletion, and sequence amplification, among others [[17], [18], [19], [47]].

#### 3.1.1 Exosomes and meningiomas

Exosome content differs between meningioma patients and healthy individuals. Ricklefs et al. discovered that circulating exosome levels were higher in meningioma patients than healthy individuals and were associated with the histopathological malignancy grade. Compared to healthy controls, the mean concentrations were 2.4 times higher in M1, 3.7 times higher in M2, and 4.5 times higher in M3. Additionally, EV concentration dropped post-surgery. Moreover, EV plasma concentrations correlated with the degree of peritumoral edema and edema, a phenomenon that was relatively significant in the MI and MII subgroups [20]. At the same time, other derived exosomes could also affect meningioma progression. For example, M2 macrophage-derived exosomes stimulated meningioma progression through the TGF- β signaling pathway [21].

Exosome-borne molecules are commonly applied in the study of meningioma diagnosis. The miR-106a-5p, miR-219-5p, miR-375, and miR-409-3p serum levels were significantly increased in meningioma patients, while the miR-197 and miR224 serum levels were significantly decreased. Therefore, these exosome-borne molecules can be used as noninvasive markers of meningioma [22]. Moreover, some molecules can be utilized beyond being used solely as meningioma staging biomarkers. Exosome-borne miRNAs such as miRNA 497 and 219 may not be solely used as meningioma biomarkers due to the correlation between miR-497 levels and benign meningioma methylation categories. Ahmed et al. found that when used together, combined biomarkers could more accurately classify Meningiomas [24,23].

Alternatively, the methylation profiling of EV-DNA could enable the correct assignment of methylation subclasses for identifying tumor-specific genome-wide copy number alterations, which could facilitate the detection of meningioma-specific mutations, including potential targeted driver mutations. Other proteins specifically associated with meningioma EVs include periostin, matrix glass protein, tendinin x, and tetrasin, all of which are usually linked to connective tissue and bone formation, implying that they could be useful in meningioma progression and invasion research [20].

#### 3.1.2 Exosomes and sellar tumors

Researchers have identified unique miRNAs within exosomes isolated from patients with PAs. Notably, these specific miRNAs appear to be associated with tumor growth, invasion, and non-hormonal effects of the disease. This discovery suggests that these miRNAs may potentially be used as biomarkers for diagnosing or developing treatments for pituitary adenomas [48]. Lyu et al. found that hsa-miR-486-5p, hsa-miR-151a-5p, hsa-miR-652-3p and hsa-miR-1180-3p are viable NFPA biomarkers using an approach based on serum exosome miRNA profiling [49]. Additionally, Zhao et al. discovered that low miR-423-5p levels can promote growth-promoting adenoma development [50]. Although few studies have explored the involvement of exosome-derived lncRNAs and circRNAs in PAs, the lncRNA H19 levels in exosomes can be used to determine the prognosis of PRLPA patients [51,52].

Matrix metalloproteinase 1 (MMP-1), N-cadherin, E-cadherin, Epcam, CDK 6, RHOU, INSM 1, and RASSF10 were found to be increased in serum exosomes in invasive NFPA patients compared with non-invasive NFPA patients. Therefore, these molecules can be used as diagnostic biomarkers for invasive Pas [[53], [54], [55], [56]]. Through, transcriptomic and proteomic analysis of variants of noninvasive and invasive pituitary adenomas and other forms of pituitary adenomas, Ren et al. demonstrated that the expression of matrix metalloproteinase-1 (MMP-1) was upregulated and its formation in exosomes was associated with the aggressiveness of nfpa, that is, MMP1 mRNA and protein were specifically localized to exoinfpa, and the MMP 1 activity was transferred to other tumor cells and vascular endothelial cells [57]. Chen et al. reported that emt-related biomarkers in exosomes are potential biomarkers for assessing the EMT tendency of pituitary adenomas and for the diagnosis of invasive pituitary adenomas [25]. Another study indicated that CDK 6 or RHOU have higher sensitivity and accuracy in distinguishing INF–PAs from NNF–PAs [58].

Pituitary adenomas are tumors that can form in the pituitary gland, a pea-sized structure located at the base of the brain. These tumors can disrupt hormone production, leading to various health problems throughout the body. In some cases, abnormal hormone levels can cause excessive tissue and organ growth, including bones. Recent research suggests that exosomes, tiny membrane sacs released by cells, may also play a role in this process. For instance, Xiong et al. discovered that hSA-miR 21-5p could be transferred from GHPA cells to osteoblasts. This leads to increased bone and trabecular formation *in vivo* through the miR-21/PDCD4/AP-1 pathway, contributing to the pathological progression of acromegaly [59].

Other cell-derived exosomes can affect PA invasion progression. In the study by Wang et al., it was observed that GT1.1 cells increased the invasive capacity of RAW264.7 cells through exosomes and promoted their differentiation into osteoclasts [60]. Other scholars have shown that TAFs (tumor-associated fibroblasts) upregulate the expression of the target gene ONECUT2 via exosomal circdn1b by sponging miR-145-5p, thereby transcriptionally regulating FGFR3, activating the downstream MAPK pathway, and enhancing the progression of PA [61].

Interestingly, there is evidence that pituitary-derived exosomes act as non-hormonal messengers that mediate intercellular communication. Zhou et al. found that GH 1-exo inhibited amino acid biosynthesis and suppressed cAMP, production leading to the reduction in the expression of Eif2ak2 and downstream Atf 4 in the liver. Through this approach, it reduced the stress response in recipient hepatocytes. It has been proposed that pituitary exosomes may transfer benign features to malignant cells, thereby suppressing the cell motility, or may alter the tumor microenvironment to mitigate metastasis [62]. Their findings also provide new directions to guide future studies on pituitary-related tumors.

#### 3.1.3 Exosomes and melanoma

Notably, previous studies focused on exploring the role and treatment of exosomes in cutaneous melanoma. For instance, melanoma stem cell exosomal microRNA-4535 have been reported to promote metastasis by inhibiting the autophagy pathway [63]. Exosomal miR-211-5p regulates the glucose metabolism, pyroptosis, and the immune microenvironment in melanoma via GNA 15 [64]. While research on exosomes and intracranial melanoma is limited, insights can be gleaned from studies on cutaneous melanoma.

#### 3.1.4 Exosomes and medulloblastoma

Currently, few studies have investigated the relationship between exosomes and medulloblastoma. It was found that exosomal miRNAs (Let-7b-5p, miR125b-5p, and miR-181a-5p) promoted the migration and invasion of MB cells *in vitro* by activating the ERK/MAPK pathway. Therefore, they can be used as biomarkers of MB invasive [65]. Jackson et al. found that exosomes produced by pulp blasts, via extracellular matrix signals and via surface-associated proteins, stimulated medulloblastoma metastasis in a EMMPRIN and MMP-2 dependent manner. Metastatic medulloblastoma cells (D458 and CHLA-01 R) release a higher quantity of exosomes compared to non-metastatic primary medulloblastoma cells (D425 and CHLA-01). Additionally, exosomes derived from metastatic medulloblastoma cells notably augment the migratory and invasive capabilities of primary medulloblastoma cells [66]. Huang et al. detected high expression of miR-130b-3p in plasma-derived exosomes from MB patients, which suppresses tumorigenesis of MB by targeting SIK1 [67].

#### 3.1.6 Exosome-based treatment of tumors of the nervous system

The use of exosomes and their cargo molecules to treat gliomas is increasingly investigated, owing to their ability to promote glioma growth and influence the tumor microenvironment. Inhibition of tumor growth and modulation of the immune response in the tumor microenvironment (tumor microenvironment, TME) are key issues in improving tumor therapy [68]. Evidence has demonstrated that exosomes can promote BBB penetration without disrupting BBB integrity. This effect may be achieved through the upregulation of LCN 2 protein expression through the JAK-STAT 3 signaling pathway, enhancing the membrane fluidity of ECs *in vitro* and *in vivo*, and promoting the endocytosis of nanomaterials [69].

##### 3.1.6.1 Exosomes from different sources can be used for glioma treatment

Microglial-derived exosomes can inhibit tumor invasion in three-dimensional globular glioma culture and can be used as a nanotherapeutic agents for glioma cells [70], In a study by Miles J et al., mesenchymal stem cell-derived exosomes were injected into a primary brain glioma tumor model, resulting in the inhibition of glioma growth [71]. Furthermore, tumor-derived exosomes are transferred to antigen-presenting cells, where the MHC-I molecules present tumor antigens to CD8^+^ T cells. This process, facilitated by exosomal MHC-I, enhances the efficacy of immunotherapy, leading to the inhibition of tumor growth and more effective tumor cell elimination [72]. NK cells released exosomes under both stationary and activated conditions, which express killer proteins (i. e., Fas ligand (FasL) and perforin) and inhibit the growth of glioma [73]. Munich Found that dendritic cell-derived exosomes with TNF superfamily ligands could promote tumor cell apoptosis [74]. Natural killer cell-derived exosomes with miR-186 have cytotoxic effects on neuroblastoma cells [75]. Exosomes derived from neutrophils carry different cytokines, immunosuppressive molecules, and stimulatory molecules, which can have either tumorigenic or anti-tumor effects [76]. Kim et al. found that ginseng-derived exosome-like nanoparticles (GENs) inhibited tumor growth and regulated the tumor microenvironment (tumor microenvironment, Immune response in the TME). It was also found that the tumor volume in the treatment group significantly decreased from 64.16 ± 34.62 mm^3^ to 24.46 ± 13.73 mm^3^. In contrast to the control group, the tumor volume increased from 55.37 ± 75.46 mm^3^ to 67.02 ± 73.8 mm^3^ [68].

Exosome therapy for glioma focuses on harnessing the molecules they carry, including miRNAs, circRNAs, lncRNAs, and others. For instance, exosomal miR-29a-3p can suppress a specific pathway (PI3K/AKT/HIF-1α) in U251 glioma cells, leading to lower levels of enzymes (PDK1 and PDK2) involved in energy production and ultimately suppressing tumor cell growth. Downregulation of Bcl-2 and upregulation of the proapoptotic marker Bax following miR-29a-3p overexpression significantly inhibited the proliferation of U251 glioma cells and promoted apoptotic cells [11]. Exosome-derived miR-124a can decrease the viability and clonogenicity of GSCs and improve the model survival rate. Overexpression of exosome-derived miR-34a inhibited GBM cell proliferation, invasion, migration, and tumorigenesis *in vitr*o and *in vivo*, but it promoted the chemosensitivity of GBM cells to TMZ by silencing MYCN [77,78].

circRNA can hinder the migration of GBM cells by modulating the SRSF1/SRSF3/PTB axis, thereby decreasing the invasion and migration capabilities of U343-R and U251-R cells [33,38]. Exosomal MIF (migration inhibitory factor) inhibited the PI3K/AKT signaling pathway by upregulating metalloprotease inhibitor 3 (TIMP 3) and enhanced the TMZ sensitivity of drug-resistant glioma cells [79]. Exosome-delivered lnc-talc promoted M2 polarization of microglia, bound to ENO 1, modulated the GBM microenvironment and reduced the sensitivity of tumors to TMZ chemotherapy [80]. The si-pdgfr β -loaded exosomes showed high capacity to inhibit glioma progression by inactivating the PI3K/Akt/EZH 2 signaling pathway [81].

The use of exosomes targeting TAMs also has potential as a therapeutic strategy for gliomas. For example, M1-derived exosomes transfected with miR-5113p, NF- κB p50 siRNA and surface-modified IL4RPep-1 (binding to IL 4 R of tam) reduced the levels of M2 cytokines and immunosuppressive cells by downregulating target genes, while increasing the levels of M1 cytokines and immune stimulated cells, triggering tam repolarization to anti-tumor M1 phenotype leading to inhibition of tumor growth. Silencing siRNA via exosomes loaded with ccr2 effectively hindered monocyte recruitment and TAM generation, ultimately expediting tumor apoptosis [[82], [83], [84]].

Furthermore, exosomes have been extensively investigated for glioma treatment. They carry a payload of molecules or drugs that directly impact glioma proliferation, modulate the tumor microenvironment, and alter drug resistance. These exosomes encompass both natural and engineered variants. In a previous study, exosomes were extracted from U87MG human glioblastoma cells, and loaded with the anticancer drug selumetinib. It was found that GBM-derived exosomes could effectively transport selumetinib to GBM tumor site and exerted anti-tumor effect [85].

The low toxicity and high specificity of small interfering RNA therapy make it a highly promising approach for treating various refractory cancers, including GBM. By employing exosome targeting, the drug's efficacy is further enhanced. The u87mg-targeted nanoplatform was constructed using Exo-An2. This nanoplatform utilized the strong u87mg-targeting capability of Exo-An2 to efficiently deliver siRNA to U87MG cells, ultimately inducing apoptosis [86]. T7-Exo/siGalectin-9 effectively delivers siGalectin-9 to GBM cells, activates TLR 7-IRF 5 pathway, polarize macrophages to M1 phenotype, enhances the phagocytosis of macrophages on GBM cells, thereby suppressing the antitumor effect of CD8 + T cells and inflammatory response [87]. Using the neutrophil-exosome (NEs-Exos) system, the administration of doxorubicin-loaded exosomes (DOX-Exos) in the glioma mouse model resulted in a significant suppression of tumor growth and an extension of overall survival [88,89].

Evidence from previous investigations have demonstrated that natural exo exhibits limited bbb-traversal ability and lack specific targeting. Targeted peptides and genetic engineering methods are used to prepare engineered exosomes to address these problems.

Utilizing a specially designed exosome containing the miR-21 sponge construct, the expression of miR-21 was suppressed in glioma cell lines U87-MG and C6. This led to reduced proliferation, increased apoptosis rates, and a significant decrease in tumor volume [90].

Using a cluster of superparamagnetic nanoparticles known as SMNC-EXO, a combination of various superparamagnetic nanoparticles is securely affixed to the exosome. This cutting-edge SMNC-EXO platform facilitates precise delivery of therapeutic agents to specific cells, guided by an external magnetic field [83]. The loaded exosomes were furnished with superparamagnetic iron oxide nanoparticles and curcumin. Furthermore, the incorporation of the Neuropilin-1 peptide, achieved through the click chemistry method, led to the development of targeted glioma exosomes, possessing both therapeutic and imaging capabilities. Experimental results showed that the modified exosomes could effectively cross the blood-brain barrier to inhibit glioma growth [71]. Wang et al. used R-EXO's good nanoability to penetrate the blood-brain barrier (BBB) and tumor homing accumulation, recombinant exosomes carrying TMZ and dihydrotanshinone (DHT), prepared R-EXO-TMZ/DHT (R-EXO-t/D) which reversed drug resistance and enhanced lesion-targeted drug delivery [91]. In the exosome-based carrier system, cPLA2 siRNA (sicPLA2) and metformin with Exos were co-loaded. Results indicated that the combination disrupted the mitochondrial energy metabolism of GBM, inhibited tumor growth and prolonged survival of subjects [92]. Bai et al. utilized focused ultrasound (FUS) to optimize the targeted delivery of exosomes in glioma treatment. These systems exhibited better performance compared to conventional exosome preparation techniques. This innovative approach substantially enhanced the precision of exosome delivery, resulting in a stronger therapeutic effect [93].

Both natural or engineered exosomes should be tested and meet safety requirements before they can be directly applied or used as a carrier platform. Firstly, exosomes and nanomaterials pass through the BBB and do not destroy the integrity of the BBB [69]. Secondly, the safety of different exosomes. Liang et al. showed the Exo-An2-siRNA have negligible side effects on normal organs through H & E staining and body weight analysis [86]. Lee et al. have also mentioned that gbm-derived exosomes loaded with selumetinib have specific antitumor effects on U87MG cells and are nontoxic to normal brain cells [85]. In a xenograft mouse model, repeated intravenous administration of NK-Exo did not lead to weight loss or liver injury. Analysis of pertinent markers (blood aspartate aminotransferase (AST), alanine aminotransferase (ALT), albumin (ALB), alkaline phosphatase (ALP), urea, creatine, and uric acid) revealed no elevation in liver function indicators, signifying that GENs posed no harm to the liver and did not induce toxicity [68,73].

##### 3.1.6.2 Related studies of exosome treatment for meningioma

Studies have demonstrated that exosomal miR-99a-3p inhibits PA cell growth by regulating the expression of Nova 1, Dtl, and Rab27b. Exosome-derived miR-99a-3p and miR-149-5p inhibit cell viability, migration, and tube formation. Therefore, exosome-derived miRNAs are potential candidates for developing treatments for IPA [[94], [95], [96]]. Zhang's study found that the prognosis of DA treatment in patients with PRL tumors was affected by changes in LncRNAH19 levels. LncRNA H19 has been shown to exert therapeutic effects by inhibiting distal tumor cells through exosomes [51]. Additionally, circDennd1b upregulates the expression of its target gene, ONECUT2, by sponging miR-145-5p. This leads to the transcriptional regulation of FGFR3 and activation of the downstream MAPK pathway, ultimately promoting the progression of PA. Notably, inhibiting ONECUT2 and TAFs has shown potential in enhancing the efficacy of clinical drugs for PA treatment. Therefore, modulating circDennd1b holds promise as a strategy for the management of PA [61].

#### 3.1.5 Exosomes can treat tumors of the nervous system

**Fig. 4:**
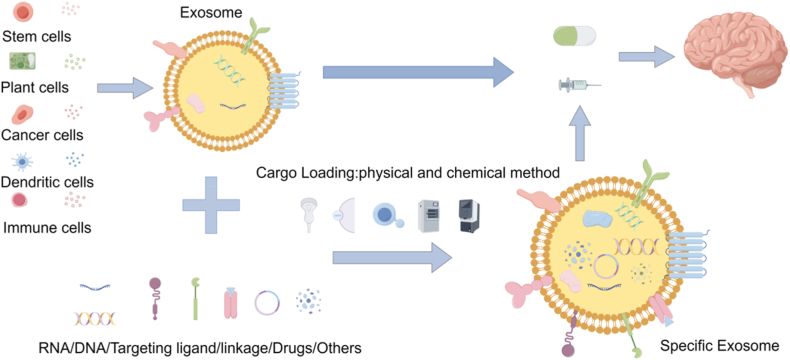
Exosomes for precision therapy of CNS tumors. Cells used for exosome extraction and production include stem cell, plant cell, cancer cell, dendritic cell, and immune cell. After extraction or production, they can be used directly for treatment, or modified to transport specific substances (DNA, RNA, drugs, etc.) for precision therapy. (By Figdrw).

The application of exosomes in tumor treatment has the following advantages: Exosomes can be extracted through *in vivo* or *in vitro* approaches, and they can act as nanocarriers of modified drugs, antibodies, and ligands. The lipid bilayer of the exosome can protect the contents, allowing them to stably stay longer in the blood circulation, without causing immunogenic reactions. More importantly, exosomes can easily cross the blood-brain barrier, which is mainly composed of brain ECs, pericytes and astrocyte end feet, which regulates the transport of molecules to the brain [97]. It has multiple protective mechanisms for preventing harmful molecules and pathogens from entering the brain microenvironment, which is also an important part of the solution of many drugs to the lesions (Fig. 4) [98,71].

#### 3.1.7 Current status and optimization of therapeutic exosomes

The challenges in utilizing exosomes for treating nervous system tumors mirror those encountered in other conditions. This approach leverages the natural properties of exosomes to create a delivery system for therapeutic agents. However, before using these exosomes in patients, scientists need to identify the ideal cell type that can both produce large quantities of exosomes and survive in conditions that promote exosome release and loading with therapeutic cargo. Current clinical trials are mainly based on cultured stem cells or immune cells, including bone marrow mesenchymal stem cells, adipose tissue-derived stem cells, monocyte-derived dendritic cells, DPSCs, DP cells, LSCs, and cancer cells [[99], [100], [101], [102]]. Furthermore, exosomes, similar to nanoparticles, are also secreted by plant organs (referred to as plant-derived exosome-like nanoparticles or PELNs). Some scientists have endeavored to isolate exosomes from plants [103,104].

The conventional clinical treatment approach encounters challenges related to low production and loading efficiency. For instance, producing less than 1 μg of exosomal protein in 1 mL of culture medium is common [105]. However, clinical treatment experiments often require 0.5–1.4 x 10^11 exosomes per individual [106]. Consequently, there is a pressing need to enhance the yield and effectiveness of exosomes through technical advancements. Therefore, it is imperative to increase the production and extraction efficiency of exosomes hence reduce the loss during processing. This challenge of mass-production of exosomes need to be addressed to promote their clinical application. Valkama suggests employing bioreactor systems, including microcarrier, hollow fiber membrane, and microfiber bed-type cultures for exosomes. This strategy to overcome the limitations of static system cultures [107]. The system offers benefits such as easy scalability, uniformity, and the ability to control culture variables, especially when integrated with online monitoring probes [108]. Exosomes cultured using 3D exhibit stronger properties, such as higher yield, higher cell proliferation, angiogenesis and immunosuppressive properties. Lee's article comprehensively examines various 3D cell culture techniques, including hanging drops, microwell arrays, scaffolds, hydrogels, rotating flasks, and the previously discussed hollow fiber bioreactors. It provides comprehensive comparison of the strengths and weaknesses of each approach (Table 2) [100].

**Table 2:** Advantages and disadvantages of different exosome cultivation methods.

Method	Advantage	shortcoming	References
Hanging drop method	1. Simple, 2. easy to implement 3.low cost 4. No support structure is required	1. Production is limited 2. Low yield	[146]
Microwell array	1. A large number of continuous production 2. With no serum conditions 3. Convenient extraction	1. Affected by the environment 2. Ball adhesion area is insufficient 3. Cell necrosis or apoptosis in the central part of the spheroid	[146] [191]
Scaffold	1. Better tissue regeneration active 2. Resistance to cellular and physiological gradients 3. The mesh scaffolds can enhance cell adhesion, proliferation, and uniform distribution of transplanted cells	/	[146]
Spinner flask	1. High yield 2. Large-scale cultivation	1. Cell trauma 2. Serum is required to form the spheroids	[146]
Fiber bioreactor	1. High yield 2. Can automatically inject nutrition and oxygen, reduce labor 3. Suitable for the automated production of large-volume exosomes	/	[146] [192]

#### 3.1.8 To improve the loading efficiency of the exosome

Exosomal cargo loading is performed using two approaches: direct exosome loading and indirect loading based on parental cells, also known as preloading and post-loading [109]. Various physical methods, such as incubation, ultrasound, extrusion, freeze-thaw cycles, electroporation, surface treatment, low permeability, and pH gradient, were investigated by Haney et al. In their study, the researchers tested four methods to incorporate catalase enzyme into exosomes. They found that sonication and extrusion were the most successful methods [110]. The chemical methods mainly include transfection (fat transfection) and in situ synthesis. Polymer nanoparticles have improved stability and higher payload capacity in drug delivery [111]. Therefore, it has been applied to investigate hybrid exosomes. Goh et al. used cell-derived nanovesicles combined with liposomes to formulate EXOPLEXs as a chimeric drug delivery platform for delivering doxorubicin hydrochloride. They reported a loading efficiency greater than 65 % [112].

#### 3.1.9 To optimize the targeting or efficacy

Exosome targeting and selective entry into recipient cells are influenced by transmembrane proteins of exosomes [113]. Artificial modification strategies have been used to perform surface or genetic modifications to improve their cell or tissue targeting specificity. Techniques such as genetic engineering, metabolic engineering, adsorption modification, and chemical modification are utilized to achieve these modifications. These methods offer versatile tools for refining and customizing exosomes to meet the requirements of specific applications. Genetic engineering fuses gene sequences of a guide protein or polypeptide with gene sequences of selected exosome membrane proteins. Metabolic engineering leverages on cellular endogenous synthesis to load target cargo into exosomes. Adsorption modifications refer to the utilization of surface-specific proteins, lipid head groups, or negative surface charges for adsorbing specific molecules [114]. On the other hand, chemical modifications involve the use of natural or synthetic ligands through coupling reactions or lipid assembly [115]. It is worth noting that surface modification can also enhance the yield of exosomes [116].

Furthermore, to mitigate the swift clearance of exosomes and enhance their duration within the affected area, researchers propose the use of bioactive scaffolds for exosome delivery. This approach can prolong the release of exosomes, potentially optimizing their therapeutic effects. These scaffolds include hydrogels, nanocomposite hydrogels, nanofiber hydrogels, and 3D printing, among others. Previously, Jiang et al. prepared chondrocyte-derived ECM stents loaded with cord msc-derived exosomes for rabbit knee cartilage defect repair. Their results demonstrated that the combined ECM + exosomes for cartilage repair showed better performance compared with exosomes or ECM stent group alone [117]. The integration of MSC-derived exosomes with collagen/chitosan via 3D printing technology yields scaffolds with excellent mechanical properties and biocompatibility. The resulting 3D–CC–BMExos scaffolds exhibit a porous network structure and high porosity, promoting cell adhesion. Therefore, they offer significantly higher cumulative release of exosomes compared to CC-BMExos scaffolds [118].

#### 3.1.10 Identify alternative sources

The aforementioned methods primarily utilize stem cells or immune cells. However, there have been investigations into utilizing exosomes derived from other organisms as an alternative to conventional cell sources. Notably, Ju et al. previously explored the extraction of therapeutic vesicles from plants [119]. Plant exosomes, also known as PELNs as mentioned in the preceding paper, offer a significant advantage due to their abundant natural sources, which partially addresses the issue of exosome yield. Studies have demonstrated that plant-derived peln has functional and morphological similarities to its mammalian counterparts [120]. It can be administered into mammals through various routes such as oral, parenteral, endotracheal, intranasal, and local administration. It is absorbed by mammalian cells through direct fusion with the plasma membrane, and via processes like phagocytosis, microcytosis, clathrin-mediated endocytosis, and lipid raft-mediated endocytosis. Compared to traditional exosomes, it offers advantages including increased safety, lower immunogenicity, and higher circulation availability. This may decrease the risk of developing chronic diseases like cancer and harmful inflammation [[121], [122], [123]]. Several plant-derived exosomes have been applied in the treatment of neurological diseases. For example, Xu et al. proposed that ginseng-derived exosomes may differentiate bone marrow-derived stem cells into functional neural cells by transferring mirna [124]. Elsewhere, exosomes isolated from carrot inhibited oxidative stress in neuroblastoma cells. In addition, peln contains large amounts of pharmacologically active molecules and nutrients that are beneficial to the receptor itself [125].

## 4 Conclusion and outlook

Exosomes carry mRNA, miRNA, lncRNA, circRNA, protein, and DNA. These molecules can be used as biological markers of central nervous system tumors to facilitate early diagnosis and initiation of early intervention and treatment. Several biomolecules contribute to the development, invasion, metastasis, and drug resistance of central nervous system tumors. Moreover, exosomes serve as natural carriers for specific molecules or drugs, making them a potential tool for the treatment of nervous system tumors. Exosomes hold immense potential for treating CNS tumors due to their versatility. Researchers should investigate their biological roles and cargo molecules in CNS tumor development, with the aim of translating these findings into diagnostic and therapeutic tools. Thus, we anticipate that the full potential of exosomes and their cargo molecules will be exploited for future clinical applications. Leveraging exosomes as a natural carrier holds tremendous promise for significant advancements in the diagnosis and treatment of various conditions, ultimately benefiting a wide range of patients.

Exosomes hold immense potential for treating central nervous system (CNS) diseases, but several key challenges need to be tackled before widespread clinical application becomes a reality. These include: (1) Developing efficient methods for exosome preparation, (2) Evaluating the specific functions of different exosomes to improve treatment targeting, (3) Assessing the therapeutic efficacy of exosomes in disease models, (4) Designing and optimizing exosome delivery systems for efficient CNS targeting. By addressing these critical areas through focused research and potential technological breakthroughs, we can pave the way for exosomes to become a powerful therapeutic tool in the fight against CNS diseases.

## Data availability statement

No data was used for the research described in the article.

## CRediT authorship contribution statement

**Mengjie Wang:** Writing – original draft, Visualization, Supervision, Methodology, Data curation. **Feng Jin:** Writing – review & editing, Funding acquisition. **Xiaoguang Tong:** Writing – review & editing, Resources, Methodology.

## Declaration of competing interest

The authors declare that they have no known competing financial interests or personal relationships that could have appeared to influence the work reported in this paper.

## Figures and Tables

**figs1:**